# Giftedness identification and cognitive, physiological and psychological characteristics of gifted children: a systematic review

**DOI:** 10.3389/fpsyg.2024.1411981

**Published:** 2024-11-20

**Authors:** Elizaveta Kuznetsova, Anastasiia Liashenko, Natalia Zhozhikashvili, Marie Arsalidou

**Affiliations:** ^1^Faculty of Educational Sciences, University of Helsinki, Helsinki, Finland; ^2^Pedagogy and Medical Psychology Department, Institute of Psychology and Social Work, Sechenov University, Moscow, Russia; ^3^Laboratory for Cognitive Research, HSE University, Moscow, Russia; ^4^Faculty of Graduate Studies, York University, Toronto, ON, Canada

**Keywords:** giftedness, intelligence, children, intelligence tests, cognitive abilities

## Abstract

Despite the extensive history of investigation, characterization and diagnostics of giftedness is still a point of debate. The lack of understanding of the phenomenon affects the identification process of gifted children, development of targeted educational programs and state of research in the field of gifted education. In the current systematic review, we seek to delineate the specific aspects in which gifted children differ from their typically developing peers in cognitive abilities, psychophysiology and psychological characteristics. Secondly, we aim to document the prevalence and criteria of intelligence tests used to assess gifted children and adolescents. We reviewed 104 articles from more than 25 countries that examined a total of 77,705 children ages 5–18 years. Results reveal a discernible trend toward adopting more culturally appropriate measures for assessing giftedness in children. Findings highlight that gifted children generally outperform their peers in several cognitive domains such as verbal working memory, inhibition, geometric problem solving, attention-switching and elemental information processing, showcasing an accuracy-reaction time trade-off. Psychophysiological assessments demonstrate heightened and accelerated brain activity during complex effortful cognitive processes. Psychological and behavioral measures reveal that gifted children score higher on tests measuring intrinsic motivation, self-efficacy, and openness to experience; as well as achieving higher grades in school and employing better problem-solving strategies. Our systematic review can be beneficial in educational and research contexts, giving directions in assessment of giftedness and designing future research.

## Introduction

Superior cognitive abilities or ‘giftedness’ have attracted the interest of philosophers (Comte, 1844; Saint-Simon, 1813), educators (Gardner, 2004; Harris, 1867), psychologists, psychiatrists (Galton, 1870; Lombroso, 1895; Renzulli, 1978), economists (Bui et al., 2011) and neuroscientists (Geake, 2008; Geschwind and Galaburda, 1985) for a long time. Despite the extensive history of investigation, characterization and diagnostics of giftedness is still a point of debate: components of giftedness and criteria for outstanding abilities lack operationalization and precision (Robinson et al., 2000; Sternberg and Davidson, 2005; Subotnik et al., 2011). While traditional views linked giftedness closely with high IQ scores, contemporary theories understand it as a multifaceted phenomenon influenced by both cognitive and non-cognitive factors. Renzulli’s Three-Ring Conception (Renzulli, 2011) suggests that giftedness arises from the interaction of above-average ability, creativity, and task commitment. Gagné’s Differentiated Model of Giftedness and Talent (Gagné, 2004) distinguishes between innate natural abilities (gifts) and systematically developed skills (talents), emphasizing the influence of environmental and personal factors in transforming gifts into talents. Sternberg’s Triarchic Theory (Sternberg, 1985) proposes that giftedness involves a balance of analytical, creative, and practical intelligences, indicating that traditional intelligence tests may not fully capture an individual’s capabilities. Nevertheless, till now intelligence tests serve as a major giftedness detection tool used by researchers and educators (Hodges et al., 2018), especially when selecting for educational programs. Besides not considering more comprehensive approach to giftedness, overall intelligence tests criteria and thresholds are not well-determined. Such practices differ among countries, states, and even educational institutions (e.g., Bélanger and Gagné, 2006; Mcclain and Pfeiffer, 2012). Cutoff criteria are typically based on either a percentage of children (e.g., the top 5% or 1%) or specific test scores (e.g., an IQ of 130 on classic IQ tests). This variability in selecting gifted children makes it challenging to compare and compile research data, thereby impacting the state of the art in the field. Gifted education is one of the areas that is closely connected with intelligence testing. Thus, lack of consistency in testing criteria affects the identification process of gifted children and the development of educational programs to accommodate the specific needs of gifted children (Silverman and Gilman, 2020; Subotnik et al., 2011).

Furthermore, understanding of giftedness imply uncovering its manifestations in cognitive abilities, motivation, personal traits, and other areas (Berg and McDonald, 2018; Topçu and Leana-Taşcılar, 2018). Nevertheless, it is still unclear whether some characteristics contribute more to the concept of giftedness and what underlying factors are responsible for them. The literature on the traits distinguishing gifted children from those with average abilities is rife with ambiguity. For example, Arffa (2007) suggested superior levels of inhibition in gifted children, while other studies do not support these findings (Duan et al., 2010; Rocha et al., 2020). Vogl and Preckel (2013) demonstrated that higher cognitive ability was correlated with increased social self-concept of assertiveness, whereas Kroesbergen et al. (2015) found no difference in social acceptance and self-concept between gifted and typically developing children. Additionally, Casino-García et al. (2021) revealed even lower scores on family, social, and physical self-concept in gifted children. The literature on mathematical skills (Giofrè et al., 2014; Paz-Baruch et al., 2022) and wellbeing (Košir et al., 2016; Kroesbergen et al., 2015; Vogl and Preckel, 2013) in gifted and mainstream children also contains significant ambiguity.

In the current systematic review we aim to provide greater clarity on what constitutes intellectual giftedness. We seek to delineate the specific aspects in which gifted children differ from their typically developing peers. Secondly, we aim to investigate which tests are most commonly used and what criteria are employed to differentiate between gifted children and their control peers to clarify the trend for practitioners and researchers.

To answer these questions in our systematic review we set the following goals:

- to document the prevalence of intelligence tests used as selection tool for gifted and control groups in children and adolescents younger than 18 years;
- to discuss comparisons between gifted and control children focusing on cognitive functions, psychophysiology, and psychological characteristics;
- to highlight existing methodological, conceptual, and reporting gaps in current research for gifted children.

This synthesized knowledge can be beneficial in educational and research contexts, giving directions in assessment and understanding of giftedness and designing future research.

## Methods

### Review design and eligibility criteria

The objectives of this review was to synthesize current evidence on the cognitive domains, psychophysiological patterns, and psychological traits that distinguish gifted children and adolescents; and to document the prevalence and criteria of intelligence tests used in assessing giftedness among children and adolescents. The relevancy of this systematic review comes from ongoing debate surrounding the lack of a unified understanding of giftedness and gaps in identification processes. By systematically analyzing existing studies, we aim to inform educational practices and highlight areas necessitating further investigation.

A systematic review was prepared in accordance with the Preferred Reporting Items for Systematic Reviews and Meta-Analyses (PRISMA; Moher et al., 2009; Page et al., 2021) and the checklist (Hutton et al., 2015). Studies included in the final set of eligible articles adhered to the following criteria: (a) written in English; (b) participants were children and adolescents younger than 18 years; (c) participants had no pre-existing medical or psychological disorders; (d) giftedness was determined using well-established intelligence tests discussed in peer-reviewed journals; (e) the intellectual giftedness was main focus of the study; (f) publications were empirical studies such as experimental studies, observational studies, and psychometric evaluations.

Including non-English articles was not feasible without compromising the quality and accuracy of the translations. By focusing on English-language articles, we ensured that the studies included in our review are accessible to a broad international audience. Second, medical and psychological disorders can significantly influence cognitive functioning, physiological responses, and psychological wellbeing (McCutcheon et al., 2023; Snyder, 2013; Webb et al., 2016). Excluding participants with pre-existing conditions reduces variability unrelated to giftedness, enhancing the internal validity of our review and allowing for more consistent comparisons across studies. Third, we considered “well-established intelligence tests” to be standardized assessments that demonstrate strong reliability and validity (e.g., internal consistency, test–retest reliability above 0.80) in measuring intelligence or cognitive abilities, supported by extensive psychometric research. This includes evidence from test manuals (e.g., Wechsler, 2014), independent peer-reviewed studies, academic textbooks and handbooks (Flanagan and Harrison, 2022), and reviews in authoritative sources like the Mental Measurements Yearbook (Buros Center for Testing, n.d.). Eligible intelligence tests were standardized on a large, representative sample, providing normative data for accurate comparison across diverse populations. These test were commonly used in the identification and assessment of gifted children in both research and applied settings and widely discussed and critiqued in peer-reviewed academic journals, indicating acceptance within the scientific and educational communities (e.g., Watkins and Canivez, 2016). Examples of such tests are the Wechsler Intelligence Scale for Children (WISC-V), Stanford-Binet Intelligence Scales (SB5), Kaufman Assessment Battery for Children (KABC-II), Raven’s Progressive Matrices, and Woodcock-Johnson Tests of Cognitive Abilities. Forth, primary outcomes included differences in cognitive domains (e.g., working memory, inhibition, geometric problem-solving), psychophysiological assessments (e.g., ERPs, oscillatory power, BOLD signal, gray and white matter volumes), and psychological measures (e.g., motivation and self-efficacy). We did not deem eligible papers where giftedness was measured only by high performance or talent in a particular subject area studied at school. Lastly, we did not include theoretical papers or conceptual analyses, literature reviews and meta-analyses, editorials, commentaries and opinion pieces, case studies, qualitative studies without empirical data, unpublished work, book and book chapters, non-peer-reviewed articles. We did not impose any restrictions on the publication date; all studies published up to the date of our search were considered for inclusion.

### Search strategy and information sources

The literature search was conducted using Web of Science,^1^ covering literature up to February 4, 2022. Additional sources included gray literature identified through reference lists of relevant articles. The search strategy included a combination of keywords ‘cognitive’, ‘gifted’, ‘intelligence’, and ‘talent’ in title and abstract. Boolean operators “AND” and “OR” were used to refine the search. The finalized keyword string was as follows:

(cognitive[Abstract] AND gifted[Abstract]) OR (intelligence[Abstract] AND gifted[Abstract]) OR (intelligence[Abstract] AND talent[Abstract]) OR (cognitive[Abstract] AND talent[Abstract]),

and included a filter on English-language articles. We did not consult with a librarian, as coauthor MA has extensive experience in conducting systematic reviews and played a key role in developing and refining the search strategy.

### Study selection and data extraction

This search yielded a total of 1,214 articles, which underwent a series of selection criteria. After duplicates were removed, studies were first screened based on their title and abstract by three authors (AL, EK, NZh). To increase the inter-rater reliability (IRR), a third of papers was independently assessed by three reviews (Al, EK and NZh). After comparing the assessment results, the IRR was high (>80%). The rest of the papers were split between those reviewers. Reviewers were free to leave the papers uncategorized due to some doubts concerning inclusion criteria. At the end of the screening uncategorized papers were examine by all three reviewers and consensus was reached by discussion. Full-text articles were retrieved for further assessment. A total of 104 articles survived criteria and were included in the review. One of the main reasons for exclusion was the choice of the test to measure giftedness or the absence of it (*n* = 117). We only deemed eligible standardized intelligence tests, which have been discussed in peer-reviewed journals. No method of selection was mentioned in 81 articles. In some articles, authors recruited children that were already identified as gifted; in that case we included the paper only if the identification measure was indicated and met our criteria. The second most common exclusion reason was the type of giftedness (*n* = 40). We rejected articles which focused solely on outstanding achievements or giftedness in other areas rather than intellect (e.g., sports, art, or a particular subject at school).

**Figure 1:**
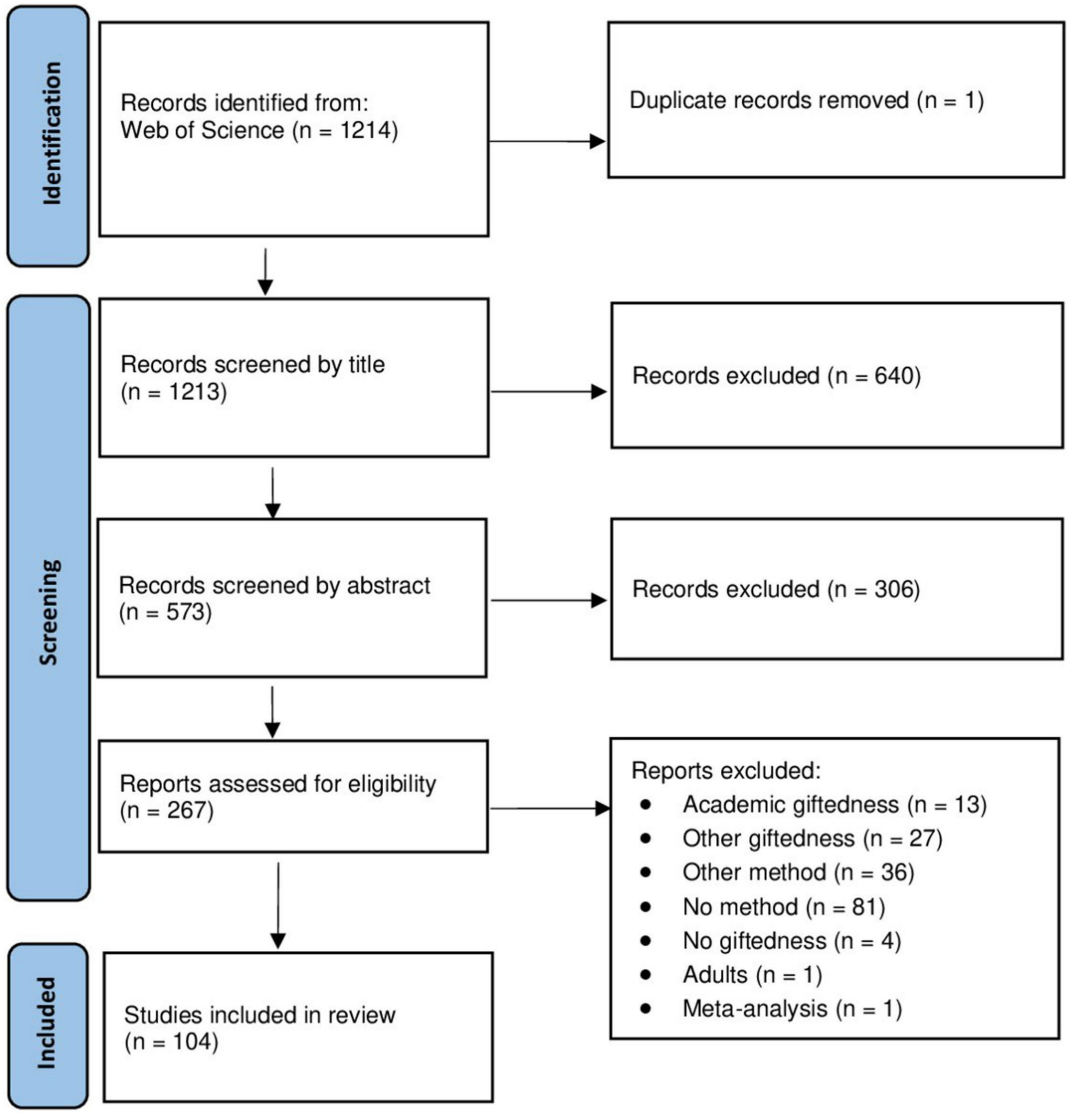
PRISMA flow diagram illustrating the study selection process for the systematic review.

A PRISMA flow chart illustrates the steps taken the study selection process, detailing the number of records identified, screened, assessed for eligibility, and included in the review (Figure 1).

Data were extracted using a standardized form capturing details on author(s), publication year, country where the study was conducted, age and gender of participants, sample size, what tests were used to select intellectually gifted children for each study, criterion of giftedness. The combined total number of participants across all relevant studies included a substantial sample of 77,705 individuals. In our systematic review we aimed to provide a comprehensive synthesis of the existing literature. To achieve this, we included studies both with and without control groups. Studies without control groups were studied to address our methodological research question concerning the intelligence tests used in identifying giftedness. Our main results regarding the characteristics of giftedness were based on studies including both gifted and control groups as it allows direct comparisons between gifted children and their non-gifted peers. Thus, if the study contained a control group we proceeded with evaluation of further methods and results, including experimental method, the target variable of analysis, the task/questionnaire chosen, and statistical differences reported between the gifted and control groups. We ensured that the control groups were demographically and contextually similar to the gifted groups in terms of age, gender, socioeconomic status, and ethnic background. This approach aimed to attribute observed differences specifically to giftedness. The combined total number of participants in articles with a control group included 27,309 individuals. To ensure accuracy, all three reviewers cross-checked the extracted data and all the terms were taken as they were used in the original articles.

### Quality assessment and data synthesis

We conducted an informal quality assessment by critically reviewing each included study’s methodology, sample characteristics, data analysis methods and reported findings. Data from the included studies were synthesized by creating a comprehensive summary (Supplementary Table 1S), which summarizes demographic, methodological choices and research findings reported by article. We organized articles in blocks based on the topic of interest: cognitive functions, psychophysiology, psychological characteristics, and other. By organizing the data in this manner, we facilitated a systematic comparison of studies, enabling us to identify patterns, similarities, and differences across various research contexts. The summary table served as the foundation for a narrative synthesis, as the heterogeneity of study designs and outcome measures precluded a quantitative meta-analysis.

## Results

### Giftedness assessments

Supplementary Table 1S summarizes information for 104 articles, including author, year of publication, topic of interest, country, number of participants, age group, measurement of giftedness, criteria of giftedness, experimental method, target of analysis, dependent variable/s and significant differences between gifted and control children. Articles were published between 1930 and 2022. Main topics of interest were: Cognitive abilities (*n* = 42), Psychophysiological data (*n* = 16), Psychological characteristics (*n* = 20), and Other (*n* = 54). Some of the articles considered more than one topic of interest. In most articles that reported participant gender, the proportion of boys and girls in the sample was comparable (*n* = 62), girls outnumbered boys in five articles, and boys outnumbered girls in 26 articles. Most of the articles were published in the USA (*n* = 38), followed by China (*n* = 12), Israel (*n* = 9), Spain (*n* = 5), Germany (*n* = 5), South Korea (*n* = 5), Canada (*n* = 4), Denmark (*n* = 4), France (*n* = 4), Australia (*n* = 2), Iran (*n* = 3), Turkey (*n* = 2), Sweden (*n* = 2), and Netherlands (*n* = 2). Other countries produced one eligible article.

For 59 out of 104 studies the target group was primary school children (from 6 to 11 y.o.), for 45 studies—younger adolescents (from 12 to 14 y.o.), for 32—older adolescents (15–18 y.o.), for 22—younger children (<6 y.o.). Some articles considered children from more than one age group.

**Figure 2:**
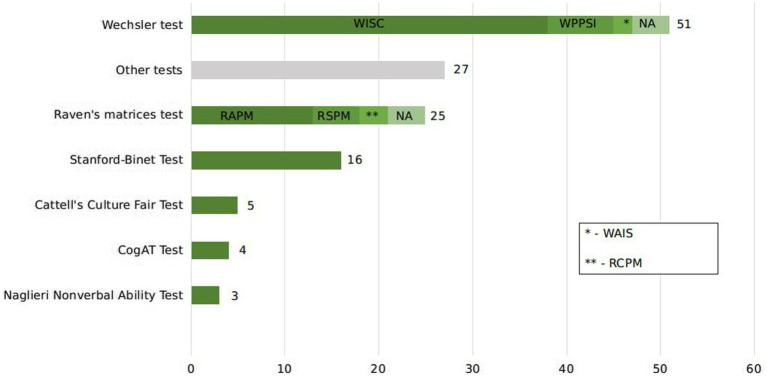
Commonly used intelligence tests for giftedness identification. CogAT Test, Cognitive Abilities Test; WAIS, Wechsler Adult Intelligence Scale; RCPM, Raven’s Colored Progressive Matrices.

Figure 2 illustrates the distributions of different tests used to select gifted and control groups in eligible articles. The Wechsler Intelligence Scale was the most popular test used (*n* = 51). Subscales of the Wechsler Intelligence Scale Test were specified, where possible. Second most popular test among eligible papers was the Raven’s Matrices Test (*n* = 25). Stanford-Binet was used 16 times, Cattell’s Culture Fair Test—five, CogAT—four and Naglieri Nonverbal Ability Test—three. Other tests (*n* = 27) were used in less than three articles and composed the category “Other tests.”

Within the category of the Wechsler scales the most frequently used test was the Wechsler Intelligence Scale for Children (WISC). The classical version of WISC was used in seven studies, the revised WISC version—in 18 studies, WISC-III—in five studies, WISC-IV—in five studies, and WISC-V in one study. The criteria of giftedness for most of the studies stayed in range from 120 to 130 (see Supplementary Table 1S, column “Criterion of giftedness”). One study set the criteria to 160 and two studies selected the top 5% and top 1% of the participants who took part in the test. The scale was used with all age groups. Another type of scale was the Wechsler Preschool and Primary Scale of Intelligence (WPPSI) which was given to younger children, primary school children, and younger adolescents in seven studies. Two papers used Wechsler Adult Intelligence Scale and administered it to older adolescents. Four papers did not specify the type of scale.

The three versions of Raven’s Progressive Matrices were used in the final sample of the review. The Advanced Progressive Matrices (RAMP; *n* = 13) were given mainly to older adolescents with the criteria of scoring higher than 26–33. The Standard Progressive Matrices (RSPM, *n* = 5) tested abilities of younger adolescents and primary school children by indicating the criteria of giftedness as top 5% or top 10% of children participated in the test. Four papers did not provide information on the criterion. The criteria for the Colored Progressive Matrices (*n* = 3) were the top 2% and standardized score more than 110 and 130. The test was given to primary school children and younger children. The other four papers did not specify the version of the Raven’s Progressive Matrices.

The criteria for the Stanford-Binet Intelligence Test (*n* = 16) mostly fell within the scores 120–135. One study set the criteria to top 5% and one study—to 150. Children from all the age groups took this test.

### Cognitive abilities

Of the 104 eligible articles, 42 examined cognitive abilities, 27 of which involved a control group. Of the 27 articles with a control group, samples of 15 studies were gender balanced, five included more males, three were exclusively male, one had more females, and three did not provide this information. One study focused on younger children, 13 studies on primary school children, 14 on younger adolescents, and nine studies evaluated older adolescents.

#### Working memory

Out of six articles that used tasks related to working memory, five of them found significant differences between gifted and control children (Table 1). Significant differences in accuracy were found in forward and backward parts of the Digit span task (Fard et al., 2016; Leikin et al., 2013), as well as in the working memory task where children had to remember words (Coyle et al., 1998) and the last item of a series (Calero et al., 2007; Zhang et al., 2017). Gifted group did not perform better than the control group in the working memory task with digits and letters and both parts of the Corsi task (Leikin et al., 2013; Paz-Baruch et al., 2016).

**Table 1:** Working memory: comparisons between gifted children and control children.

First author, year	Age group	Criterion of giftedness	Task/questionnaire	Significant differences
Fard et al. (2016)	Younger adolescents	Wechsler test, score > 110	Digit span task (forward, backward)	Accuracy higher in gifted
Calero et al. (2007)	Primary school	The Kaufman Brief Intelligence Test > 136	Last item recalling	Accuracy higher in gifted
Zhang et al. (2017)	Younger adolescents	Stanford-Binet Test, WPPSI-R, RSPM Score in top 5%	Recall task	Accuracy higher in gifted
Coyle et al. (1998)	Primary school	WISC-R, Short-form WISC-III, Score > 130	Multitrial recall task	Accuracy higher in gifted
Leikin et al. (2013)	Older adolescents	RAPM > 27	Digit span task (forward, backward)	Accuracy higher in gifted
			Letters and digits	No differences in accuracy
			Spatial Corsi test (forward, backward)	No differences in accuracy
Paz-Baruch et al. (2016)	Older adolescents	RAPM > 27	Spatial Corsi test (forward, backward)	No differences in accuracy

WPPSI, Wechsler Preschool and Primary Scale of Intelligence; RSPM, Raven’s Standard Progressive Matrices; WISC, Wechsler Intelligence Scale for Children; RAPM, Raven’s Advanced Progressive Matrices.

#### Attention

Three articles found differences in various types of attention (Table 2). Gifted children performed significantly better on the d2 test of selective attention (Paz-Baruch et al., 2016). Higher scores in accuracy for gifted primary school children were found for sustained, supervisory, and divided attention, but not in focused, alertness, spatial, and switching attention (Zhang et al., 2016). At the same time gifted children were faster on attentional switching, alertness, spatial and divided attention tasks but not on focused, sustained and supervisory attention tasks (Zhang et al., 2016). In another study, both groups performed at a high level in terms of accuracy on attentional switching task, and gifted children demonstrated shorter reaction time in comparison with controls (Duan and Shi, 2014).

**Table 2:** Attention: differences between gifted children and their control children.

First author, year	Age group	Criterion of giftedness	Task/questionnaire	Significant differences
Paz-Baruch et al. (2016)	Older adolescents	RAPM > 27	d2 test of selective attention	Higher accuracy in gifted
Zhang et al. (2016)	Primary school	RAPM > Level 1, Cattell’s Culture-Fair Test (for verification)	Focused attention identification task	No differences in accuracy No differences in reaction time
			Sustained attention number task	Accuracy higher in gifted No differences in reaction time
			Supervisory attention star counting test	Accuracy higher in gifted No differences in reaction time
			Alertness task	No differences in accuracy Shorter reaction time in gifted
			Spatial location detecting task	No differences in accuracy Shorter reaction time in gifted
			Divided attention dual visual and acoustic task	Accuracy higher in gifted Shorter reaction time in gifted
			Switching location detection task	No differences in accuracy Shorter reaction time in gifted
Duan and Shi (2014)	Younger adolescents	RSPM	Switching task	No differences in accuracy Shorter reaction time in gifted

RAPM, Raven’s Advanced Progressive Matrices; RSPM, Raven’s Standard Progressive Matrices.

#### Inhibition

Three articles out of four found differences on inhibition between the groups (Table 3). Johnson et al. (2003) found differences in processing speed between groups with high and average levels of intelligence on the Spatial location task, the Stroop task, and Trail making test where gifted children were faster in giving their responses. Gifted primary school children made less errors in the ignored repetition condition and the Stroop condition in the Stroop task. Significant differences in reaction time but not in accuracy were observed between the groups in effortful inhibition measured by the Trail making test and automatic inhibition measured by the spatial location task. Montoya-Arenas et al. (2018) research did not find significant differences in accuracy on the Stroop task, no reaction time scores were reported. Younger adolescents scored higher and reacted faster in cognitive control and conflict control tasks (Liu et al., 2011a, 2011b).

**Table 3:** Inhibition: differences between gifted children and their control children.

First Author, year	Age group	Criterion of giftedness	Task/questionnaire	Significant differences
Johnson et al. (2003)	Primary school	WISC-III (Grades 1–3) > top 1%, WISC-III (Grades 4–5) > top 3%, CCAT, Canadian Achievement Test-2	Automatic inhibition spatial location task	No differences in accuracy Shorter reaction time in gifted
			Effortful inhibition trail making test	No differences in accuracy Shorter reaction time in gifted
			Stroop task	Higher accuracy in gifted Shorter reaction time in gifted
Montoya-Arenas et al. (2018)	Primary school	WISC-III > 130	Stroop task	No differences in accuracy
Liu et al. (2011b)	Younger adolescents	WPPSI, Stanford–Binet Test	Eriksen flanker task	Higher accuracy in gifted Shorter reaction time in gifted
Liu et al. (2011a)	Younger adolescents	WPPSI	Go-nogo task	Higher accuracy in gifted No differences in reaction time

WISC, Wechsler Intelligence Scale for Children; WPPSI, Wechsler Preschool and Primary Scale of Intelligence.

#### Abstract reasoning and planning

Both groups of primary school children showed comparable time and numbers of moves in solving the Tower of Hanoi/London (Montoya-Arenas et al., 2018; Vogelaar et al., 2019; Table 4). In the mental rotation task gifted younger and older adolescents showed higher accuracy, whereas there was no significant difference in reaction time between the groups (Anomal et al., 2020). Results for the Wisconsin Card-Sorting Test that covers a set of executive functions demonstrate the controversy: significant differences between gifted children and control children were found in one article (Tanabe et al., 2014) but not another (Montoya-Arenas et al., 2018). Researchers found higher accuracy of the gifted group in geometry problem solving (Vogelaar et al., 2017; Waisman et al., 2016), and inductive reasoning task (Zhang et al., 2015).

**Table 4:** Abstract reasoning and planning: differences between gifted children and control children.

First author, year	Age group	Criterion of giftedness	Task/questionnaire	Significant differences
Anomal et al. (2020)	Younger adolescents Older adolescents	WISC, WAIS Score > 120	Shepard-Metzler mental rotation task	Accuracy higher in gifted No differences in reaction time
Vogelaar et al. (2019)	Primary school	RSPM in top 5%	Tower of London	No differences in number of moves
Montoya-Arenas et al. (2018)	Primary school	WISC-III > 130	Tower of Hanoi	No differences in number of moves
			Wisconsin Card-Sorting Test	No differences in number of categories achieved
Tanabe et al. (2014)	Younger children Primary school Younger adolescents Older adolescents	WISC-IV > 130	Wisconsin Card-Sorting Test	More conceptual-level responses in gifted
Zhang et al. (2015)	Older adolescents	RAPM > 32	Reasoning task	Accuracy higher in gifted
Vogelaar et al. (2017)	Primary school	RSPM in top 10%	Geometric analogy problem solving	Accuracy higher in gifted
Waisman et al. (2016)	Older adolescents	RAPM > 26	Geometry problem solving	Accuracy higher in gifted

WISC, Wechsler Intelligence Scale for Children; WAIS, Wechsler Adult Intelligence Scale; RSPM, Raven’s Standard Progressive Matrices; RAPM, Raven’s Advanced Progressive Matrices.

#### Elementary cognitive processes

Elementary cognitive tasks are often basic tasks that pose limited requirements on cognitive processes that are not commonly considered as working memory, attention, and inhibition tasks. In elementary processing tasks (Table 5), the gifted groups showed better results in cross-out of numbers that measured the speed of processing (Paz-Baruch et al., 2014) as well as in choice reaction time task (Duan et al., 2013) but not in other speed of processing tasks such as the visual matching test, the digit-symbol test, and the symbol search test (Paz-Baruch et al., 2014, 2016). Investigating speed and efficiency of elemental information processing, Kranzler et al. (1994) detected an advantage of gifted younger adolescents in reaction time in the simple reaction time and odd-man paradigm tasks but not in the choice reaction time task. Notably, the authors also analyzed movement time, the interval between releasing the home button and depressing the push button, which resulted in showing differences between the groups in all three tasks. In line with this study, Duan et al. (2013) reported differences in the speed of information processing between gifted and control primary school children using perceptual and processing tasks. They used several measures such as the inspection time task (deciding which stimulus line is longer), the choice reaction time task (judging whether the sample stimulus appeared in the line of other stimuli), and the abstract matching task (choosing appropriate patterns based on the sample one). For all three measures, gifted children were quicker than those in the control group.

**Table 5:** Elementary cognitive processing: differences between gifted children and control children.

First author, year	Age group	Criterion of giftedness	Task/questionnaire	Significant differences
Paz-Baruch et al. (2014)	Older adolescents	RAPM > 26	Cross-out of numbers	Accuracy higher in gifted
			Visual-matching	No differences in accuracy
			Digit-symbol test	No differences in accuracy
			Symbol-search	No differences in accuracy
Paz-Baruch et al. (2016)	Older adolescents	RAPM > 26	Visual-matching	No differences in accuracy
			Digit-symbol test	No differences in accuracy
			Symbol-search	No differences in accuracy
Duan et al. (2013)	Primary school Younger adolescents	Cattell’s Culture Fair Test in top 5%	Inspection time task	Shorted reaction time in gifted
			Choice reaction time task	Higher accuracy in gifted Shorted reaction time in gifted
			Abstract matching (shape discrimination) task	Higher accuracy in gifted Shorted reaction time in gifted
Kranzler et al. (1994)	Younger adolescents	RAPM	Simple reaction time task	Shorted reaction time in gifted Shorter movement time in gifted
			Choice reaction time task	No differences in reaction time Shorter movement time in gifted
			Odd discrimination paradigm	Shorter reaction time in gifted Shorter movement time in gifted

RAPM, Raven’s Advanced Progressive Matrices; SAT, Scholastic Aptitude Test.

#### Other

Examining self-control abilities using facial expressions, Urben et al. (2018) revealed shorter reaction times of average ability children for neutral and happy faces but no difference for sad faces. Higher accuracy for gifted children in the tasks related to memory and learning was found in auditory verbal learning (Fard et al., 2016), the learning potential test (Calero et al., 2011), and metacognitive competences (Tibken et al., 2022). Researchers also revealed higher skills of the gifted group in reading comprehension (Vogelaar et al., 2017), verbal fluency (Montoya-Arenas et al., 2018), fluid intelligence measured by RSPM (Li et al., 2020), concrete and formal operations (Carter and Ormrod, 1982) and strategic thinking (Coyle et al., 1998; Yun et al., 2011) in comparison with their peers. Higher accuracy in gifted children was observed in mental-attention capacity (Johnson et al., 2003), pattern recognition task (Paz-Baruch et al., 2016), self-regulation and concentration (Calero et al., 2007), and simple arithmetic exercises that measured the speed of processing (Paz-Baruch et al., 2014).

#### Psychophysiological data

Sixteen articles examined psychophysiological data, 14 of them focused on differences between gifted and control groups.

##### Event related potentials (ERP) studies

There were eight articles investigating event-related potentials (ERPs) related to cognitive functions (Table 6). Six of them demonstrated significant differences between gifted and control children in at least one ERP component.

**Table 6:** ERPs: comparisons between gifted children and control children.

First author, year	Age group	Criterion of giftedness	Event-related potentials (task)	Significant differences
Liu et al. (2015)	Younger adolescents	Cattell’s Culture Fair Test	P1 (facial expression identification)	No differences in amplitude
Waisman et al. (2016)	Older adolescents	RAPM >26	P1 (geometric problems solving)	Higher amplitude in gifted Longer latency in gifted
Liu et al. (2011b)	Younger adolescents	WPPSI, Stanford–Binet Test	N2 (Eriksen flanker task)	Higher amplitude in gifted
			N2 (emotional face-word Stroop task)	Higher amplitude in gifted
			P3 (Eriksen flanker task)	Higher amplitude in gifted Shorter latency in gifted
Li et al. (2020)	Primary school	RSPM	N2 (Emotional Simon task)	Higher amplitude in gifted Shorter latency in gifted
Liu et al. (2011a)	Younger adolescents	WPPSI	P3 (GoNogo task)	Higher amplitude in gifted
			Cue-P3 (GoNogo task)	Higher amplitude in gifted
Duan and Shi (2014)	Younger adolescents	RSPM	P3 (attention switching task)	No differences in amplitude No differences in latency
Liu et al. (2007)	Primary school Younger adolescents	Stanford–Binet Test (revised), WPPSI-R	P3a (Involuntary attention switching task)	Higher amplitude in gifted
			MMN (Stimulus discrimination task)	Higher amplitude in gifted
			Late discrimination negativity (Stimulus discrimination task)	Higher amplitude in gifted
Anomal et al. (2020)	Younger adolescents Older adolescents	WISC, WAIS Score > 120	Rotation-related negativity (Shepard-Metzler mental rotation task)	Higher amplitude in gifted (right hemisphere)

WPPSI, Wechsler Preschool & Primary Scale of Intelligence; RAPM, Raven’s Advanced Progressive Matrices; RSPM, Raven’s Standard Progressive Matrices; WISC, Wechsler Intelligence Scale For Children; WAIS, Wechsler Adult Intelligence Scale.

One out of two articles analyzing the P1 component of ERPs (peaks ~100 ms after stimulus onset) found a significant difference between the gifted and the control group. Amplitude of the parietal P1 was higher in gifted adolescent boys when solving geometric problems (Waisman et al., 2016). Gifted children also experienced the peak of P1 later than the control group, in other words, the latency of P1 in this study was higher in gifted children (Waisman et al., 2016). Another paper, a facial expression identification study, revealed no difference in P1 response between gifted and control groups of younger adolescent boys during the early visual processing stage (Liu et al., 2015).

Both articles investigating the N2 component of ERP (~200–350 ms post-stimulus) found a significant difference between the gifted and the control groups. The N2 component had larger amplitude in gifted children for both classic conflict imposed by the Eriksen flanker task (Liu et al., 2011b) and emotional conflict by face-word Stroop task (Liu et al., 2011b) and by emotional Simon task (Li et al., 2020). The emotional Simon task also revealed lower N2 latency in gifted children (Li et al., 2020).

Three out of six articles investigating ERPs analyzed the P3 component (roughly 250–500 ms after stimulus onset). Two articles revealed increased Р3 amplitude in gifted children, both in primary school students and younger adolescents, both in response to a stimulus and in response to a cue providing information about future stimulus (Liu et al., 2011b, Liu et al., 2011a; Zhang et al., 2006). One out of these three articles showed that gifted children demonstrate shorter latency of the P3 component (Liu et al., 2011b; Zhang et al., 2006). However, no difference was found neither in P3 amplitude nor in latency in the study of Duan and Shi (2014) who investigated attentional switching performance in younger adolescents. One article focused on the P3a (~250–280 ms after stimulus onset, mostly associated with the processing of novelty) and the mismatch negativity components (200–400 ms). For both components, a larger amplitude in gifted primary school students and younger adolescents was observed (Liu et al., 2007). One study investigated mental rotation-related negativity (~400 ms post-stimulus onset) and the late discriminative negativity (400–700 ms; Anomal et al., 2020; Liu et al., 2007). For both components greater amplitudes in gifted primary school students and younger adolescents was observed. Overall, gifted children tend to show greater ERP amplitudes during problem solving.

##### Electroencephalography (EEG) oscillations studies

Two articles examined brain oscillations using EEG. Both articles investigating EEG oscillations related to cognitive functions show significant differences between gifted and control children in gamma frequency band.

When performing easy tasks for reasoning, gamma rhythm power (30–45 Hz) was lower in gifted older adolescents, whereas when performing difficult tasks, gifted children exhibited significantly increased gamma power compared with a control group (Zhang et al., 2015). Only the gifted group showed a significant increase in gamma power with task difficulty (Zhang et al., 2015). Zhang et al. (2014) analyzed temporal binding of the gamma-band (30–60 Hz) synchronization between frontal and parietal cortices in adolescents with exceptional mathematical ability. Compared with the average-ability participants, the math-gifted adolescents showed a highly integrated fronto-parietal network due to distant gamma phase-locking oscillations. Gifted adolescents also demonstrated more stable frontal–parietal gamma phase dynamics (Zhang et al., 2014).

##### Other physiological methods

One article examined functional Magnetic Resonance Imaging metrics with the aim to investigate the neural bases for intellectual giftedness in adolescents. The authors showed that the blood-oxygen-level-dependent (BOLD) signal in the posterior parietal cortex was significantly stronger in gifted older adolescents than in their control peers (Lee et al., 2006). Two articles investigated Diffusion Tensor Imaging (DTI) indicators. Such topological characteristics of the brain network as levels of global and local efficiency were higher in gifted older adolescents (Ma et al., 2017). Axial diffusivity, reflecting white matter integrity, was higher in gifted primary school students and young adolescents (Nusbaum et al., 2017). Another article focused on skin conductance in response to an orientation reflex, which was increased in gifted primary school students (Kimmel and Deboskey, 1978).

### Psychological characteristics

Twenty articles examined psychological characteristics, 12 of which focused on differences between gifted and control children (Table 7). Four articles were devoted to motivation characteristics of gifted children. Three of them showed higher scores of intrinsic motivation in the gifted group (Bergold et al., 2020; Gottfried and Gottfried, 1996; Guez et al., 2018). One article investigated achievement motivation in older adolescence and found it to be enhanced in the gifted group (Wirthwein et al., 2019). One article focused on extrinsic motivation and demonstrated significantly higher scores in gifted younger adolescents (Guez et al., 2018).

**Table 7:** Motivation: comparisons of gifted children and control children.

First author, year	Age group	Criterion of giftedness	Questionnaire	Significant differences
Bergold et al. (2020)	Older adolescents	Short version of the revised Culture Fair Intelligence Test Scale 2 > 130	Investigative vocational interests (General Structure of Interests Tests)	Higher in gifted
			Intrinsic motivation in math (The intrinsic value subscale of the Scale for the Assessment of Subjective School Related Task Values)	Higher in gifted
Gottfried and Gottfried (1996)	Primary school Younger adolescents	WISC-R > 130	Intrinsic motivation (Children’s Academic Intrinsic Motivation Inventory [CAIMI])	Higher in gifted
Wirthwein et al. (2019)	Older adolescents	Intelligence-Structure-Test +2,000 R > 120	Achievement motivation (Achievement Motives Scale [German version])	Higher in gifted
Guez et al. (2018)	Younger adolescents	Chartier’s Reasoning Test on Playing Cards >130	Intrinsic motivation (Academic Self-Regulation Questionnaire)	Higher in gifted
			Extrinsic motivation (Academic Self-Regulation Questionnaire)	Higher in gifted

WISC-R, Wechsler Intelligence Scale for Children Revised.

Three articles investigated psychological characteristics related to self-efficacy (Table 8). Academic self-efficacy (Guez et al., 2018), math ability self-concept (Bergold et al., 2020), self-esteem intelligence scores (Wirthwein et al., 2019) and self-regulatory efficacy (Guez et al., 2018) were higher in gifted children in comparison with control children. However, there was no difference in social self-efficacy between the groups (Guez et al., 2018). One article also did not obtain a significant difference in social adaptation in children of different ages (López and Sotillo, 2009).

**Table 8:** Self-efficacy: comparisons between gifted children and control children.

First author, year	Age group	Criterion of giftedness	Questionnaire	Significant differences
Bergold et al. (2020)	Older adolescents	Short version of the revised Culture Fair Intelligence Test Scale 2 > 130	Math ability self-concept (Four modified items from the Scales for the Assessment of Academic Self-Concept)	Higher in gifted
Wirthwein et al. (2019)	Older adolescents	Intelligence-Structure-Test +2,000 R > 120	Self-esteem intelligence (7-point Likert scale)	Higher in gifted
Guez et al. (2018)	Younger adolescents	Chartier’s Reasoning Test on Playing Cards > 130	Academic self-efficacy (Children’s Perceived Self-Efficacy scales)	Higher in gifted
			Self-regulation score (Children’s Perceived Self-Efficacy scales)	Higher in gifted
			Social self-efficacy score (Children’s Perceived Self-Efficacy scales)	No differences

Two studies analyzed the Big 5 personality factors. Both of them found that gifted adolescents were significantly more open to experience and did not differ from ordinary peers in Extraversion, Agreeableness and Conscientiousness (Limont et al., 2014; Wirthwein et al., 2019). One article investigated the relationship between giftedness and perfectionism (Lavrijsen et al., 2021). Gifted younger adolescents had significantly higher scores on the multidimensional perfectionism scale, and, at the same time, lower concern over mistakes.

The level of life satisfaction did not differ between gifted adolescents and the control group (Bergold et al., 2015, 2020). One article focused on different characteristics of overexcitability (Limont et al., 2014). The authors found that gifted adolescents did not differ from their peers in emotional overexcitability, however gifted adolescents surpassed control children in other types of overexcitability (Sensual, Intellectual, Psychomotor, Imaginational).

According to four studies focusing on strategic behavior, gifted children come up with a better strategy when playing games (Chung et al., 2011; Yun et al., 2011) and solving tasks (Coyle et al., 1998; Zhang et al., 2017) than their control peers. Also gifted children tend to cooperate more, be less sensitive to loss (Chung et al., 2011) and stick to one particular strategy (Coyle et al., 1998). The monetary acceptance rate and earnings in the Ultimatum game conducted by Yun et al. (2011) was lower for gifted than control children (Table 9).

**Table 9:** Strategies: comparisons between gifted children and control children.

First author, year	Age group	Criterion of giftedness	Strategy	Significant differences
Chung et al. (2011)	Younger adolescents	WISC > 130	Cooperation	Higher in gifted
			Sensitivity to loss	Lower in gifted
Yun et al. (2011)	Younger adolescents	WISC-III	Strategic decisions ratio	Higher in gifted
			Monetary offers for unfair condition	No differences
			Monetary acceptance rate	Lower in gifted
			Earnings in the game	Lower in gifted
			Distributions of offers	No differences
Coyle et al. (1998)	Primary school	WISC-R and Short-form WISC-III > 130	Stability in memorizing strategy	Higher in gifted
Zhang et al. (2017)	Younger adolescents	Stanford-Binet, WPPSI-R and RSPM, top 5%	Clustering memorizing strategy	Higher in gifted

Creativity was measured in four studies, with only one evaluating differences between gifted and control children (Kershner and Ledger, 1985). Kershner and Ledger (1985) used the Torrance Tests of Creative Thinking to measure verbal and figural originality, fluency and flexibility. Authors found significant differences between gifted children and controls only in the verbal originality dimension.

### Other categories

Besides three main groups of studies listed above, there were four other categories we identified. Seven studies examined participants’ achievements, both during the school years and later in adult life. Four of them focused on differences between gifted and control children. According to results, gifted children have higher grades at school (Li et al., 2020; Wirthwein et al., 2019) and on important examinations (Guez et al., 2018). The probability to get a Master’s degree is also higher among gifted children in comparison with controls (Bergman et al., 2014). However, differences in future income level are noticeable only when comparing gifted and control boys, with gifted boys earning more (Table 10).

**Table 10:** Achievements: comparisons between gifted children and control children.

First author, year	Age group	Criterion of giftedness	Achievements measure	Significant differences
Li et al. (2020)	Primary school	RSPM	School grades	Higher in gifted
Wirthwein et al. (2019)	Older adolescents	Intelligence-Structure-Test >120	School grades	Higher in gifted
Guez et al. (2018)	Younger adolescents	Chartier’s Reasoning Test on Playing Cards >130	Grade on national examination	Higher in gifted
Bergman et al. (2014)	Primary school Younger adolescents Older adolescents	Swedish WIT III and DBA intelligence tests, top 10%	Master’s degree probability	Higher in gifted
	

Five studies investigated aspects related to family environment, three of them focused on differences between gifted and control children. According to Weissler and Landau (1993) parents of gifted children are less authoritarian and tend to expose their children to a wider variety of things (toys, books, works of art, traveling) and different sources of information. In general, they pay more attention to the child’s development and cultivation of intelligence. Landau and Weissler (1993) conducted the similar research in the same year and confirmed that at least fathers of gifted children are more educated and liberal, while at the same time the level of parents’ assertiveness was higher in families with gifted students. No differences were found in such dimensions as socioeconomic status, atmosphere at home, cognitive interaction between parents and children, diversity of parents’ interests and parents’ level of stress. In the third study comparing the environment in families with and without gifted children no differences were found at any of the dimensions, including democratic/authoritarian family style, achievement orientation, intellectual-cultural orientation, communication and organization (Schilling et al., 2006).

Fifteen studies focus on methodology of assessing gifted children, reporting significant correlations between different intelligence tests, and comparing consistency of results among them. None of these studies compared gifted and control group performance as such.

The last category combined studies (*n* = 27) which topics could not be included in one of the above–listed categories. There were various studies looking for an association between IQ and breakfast consumption (Hisam et al., 2015), TV comprehension (Abelman, 1995), height (Hollingworth, 1930), handedness (Aliotti, 1981), sleep behavior (Demirhan et al., 2018; Piro et al., 2021). Some of the studies revealed unexpected significant differences in gifted and intellectually average children, for example in height (Hollingworth, 1930) and TV comprehension (Abelman, 1995), while in other fields gifted children did not differ much from their intellectually average peers, e.g., in breakfast consumption (Hisam et al., 2015) and sleep behaviors (Piro et al., 2021).

## Discussion

The objective of this study was to enhance the understanding of giftedness identification, along with the cognitive, physiological, and psychological characteristics associated with gifted children. We systematically reviewed the literature to document the prevalent tests used to identify intellectual giftedness in children and synthesized data from studies comparing gifted and control children in cognitive, physiological and psychological domains. In accordance with the three questions outlined in the Introduction, we synthesize the key findings as follows: (а) although the Wechsler Intelligence Scale Test remains the most popular choice for identifying giftedness in children, there is a discernible trend, particularly from the 1990s onwards, favoring culturally independent inclusive tests with visual or non-verbal stimuli such as Raven’s Matrices; (b) Results indicate that gifted children outperform their peers in cognitive tasks related to verbal working memory, inhibition, attentional switching, geometric problem solving, and elemental information processing, while physiological studies reveal differences in brain activity and structure, showing increased activity in late components of evoked potentials and during complex problem-solving processes; furthermore, gifted individuals exhibit higher intrinsic motivation, self-efficacy, openness to experience, and better school achievements and problem-solving strategies; (c) Significant methodological, conceptual, and reporting gaps exist in current research on gifted children, including variability in measurement approaches, lack of standardization in assessment methods, controversial binary group divisions, inconsistent terminology for cognitive functions and inconsistencies in reporting styles that hinder reliable comparisons and synthesis of findings. The ensuing discussion maps assessments and attributes of children with outstanding intellectual abilities compared to their same age peers in control groups in cognitive functions, psychophysiology, psychological and behavioral characteristics. Concluding, we point to limitations in methodological practices and considerations for future research.

### Giftedness assessments

In our systematic review we demonstrate that classic intelligence tests have been used for many decades and remain popular in evaluation of superior intelligence. About 39% of studies used a version of the Wechsler Intelligence Scale test for assessing intellectual giftedness in children. We also reveal a trend for visual–spatial assessments that has become prominent in the last 30 years. The Raven’s Matrices Test is the second most popular assessment method in our systematic review: it evaluates visual–spatial abilities and minimizes cultural, and verbal confounds. It is often used to collect data in non-English speaking countries such as China, Spain, Israel, and Iran. This is consistent with reports that demonstrate an advantage of nonverbal tasks for screening and identifying gifted children from White, Hispanic and African American backgrounds (Lewis, 2001). The third most popular test is the Stanford-Binet, which was the one of the first tests to determine intellectual capacity in children and had a major influence on the future development of intelligence testing (Boake, 2002; Pichot, 1948). These findings highlight the importance of adopting more inclusive methods for identifying giftedness, as traditional approaches may inadvertently overlook the diverse talents of children from various cultural backgrounds. By prioritizing assessments that reduce verbal and cultural biases, we might begin to foster a more equitable framework for recognizing and nurturing gifted potential across all demographics. Such an approach not only broadens the definition of giftedness but also could lead to educational practices and policies that are more attuned to the needs of a diverse student population.

Notably, different studies employed varying threshold scores to define high intelligence. Most studies included the top 5–10 percent of scorers in the gifted group. Although there are methodological considerations for obtaining substantial sample sizes, variation in thresholds could potentially lead to differences in outcomes and interpretations of research results. The implications of establishing such thresholds are also profound for educators, specialists in gifted education, and program developers, as standardized criteria could enhance identification processes, inform targeted interventions that meet the specific needs of gifted children and promote equitable access to gifted programs across diverse populations. Thus, the need for a universally agreed-upon threshold for defining giftedness remains.

Giftedness involves a balance of analytical, creative, and practical intelligences (Sternberg, 1985). Therefore, despite their wide popularity traditional intelligence tests may not fully capture an individual’s capabilities. It would be beneficial to adopt a more comprehensive approach to identifying giftedness that goes beyond traditional assessments. For example, utilizing existing tools that measure creativity, and practical problem-solving abilities can provide a more complete picture. Besides, incorporating qualitative measures such as teacher evaluations, peer reviews, self-assessments, and observational data might be advantageous in providing a holistic view of a student’s abilities. Lastly, encouraging educational policymakers to revise identification criteria for gifted programs to include multiple intelligences could lead to more inclusive and effective educational strategies.

### Cognitive abilities

In this section, we summarize the findings related to the cognitive characteristics of gifted children, highlighting their significance in advancing our understanding of giftedness. By examining these results, we aim to provide insights that can inform both theoretical frameworks and practical applications in the field of gifted education.

Group performance was significantly different between gifted children and control children in cognitive tasks in about 70% of comparisons evaluating reaction time, 53% evaluating accuracy, and 59% evaluating both accuracy and reaction time. These findings suggest that reaction times may be a more sensitive metric in distinguishing between gifted and control groups in the cognitive tasks.

Considering working memory tasks, 63% of comparisons showed significant differences in accuracy between gifted and control children. Friedman et al. (2006) specifically showed that working memory is highly correlated with intelligence and showcasing the value of working memory tasks for assessing giftedness. In our review, gifted children showed better results on both forward and backward digit span task (Calero et al., 2007; Fard et al., 2016; Leikin et al., 2013), on recall of categorized words (Coyle et al., 1998; Zhang et al., 2017), as well as manipulating them (Calero et al., 2007; Fard et al., 2016; Leikin et al., 2013). However, gifted children did not show better accuracy in the Corsi task that involves visual–spatial working memory (Leikin et al., 2013; Paz-Baruch et al., 2016). Thus, on working memory tasks gifted children exhibit superior accuracy in storage, manipulation and retrieval, but not in spatial domain, specifically in the Corsi block-tapping task. This discrepancy in results may be attributed to the Corsi task’s greater cognitive demands and its motor component, while the digit span and recall tasks are typically less effortful (Piccardi et al., 2019). However, it is worth noting that the studies utilizing the Corsi task did not analyze reaction times, which may limit a comprehensive comparison between gifted and control children; if reaction times had been measured, it is possible that gifted children could have shown advantages in the spatial domain as well, as spatial attention tasks revealed more differences in reaction time than accuracy between gifted and control groups.

Results on attention revealed a trade-off pattern between speed and accuracy. Enhanced speed, rather than accuracy, has been demonstrated by gifted children in alertness, spatial attention (Zhang et al., 2016) and attentional switching (Duan and Shi, 2014; Zhang et al., 2016). While the enhanced precision at expense of superior speed was established in sustained and supervisory attention domains (Zhang et al., 2016). In divided attention gifted children outperformed their control children in both speed and accuracy (Zhang et al., 2016).

A speed-accuracy trade-off was also revealed for gifted group in both automatic and effortful inhibition tasks (Johnson et al., 2003), where they demonstrated enhanced speed rather than accuracy; and go-no-go task (Liu et al., 2011a), in which gifted children answered more accurately than their peers but demonstrate similar reaction time. Gifted children outperformed in both accuracy and reaction time in Eriksen flanker task (Liu et al., 2011b). Critically, inhibition processes that characterize Flanker tasks are considered more cognitively loaded, as competing cognitive processes need to be suppressed, whereas inhibition to the go-no-go task involves the suppression of a dominant response (Hung et al., 2018). Thus, children in the gifted group seem to show better performance on tasks with higher cognitive demands and overall tend to be either faster or more accurate in tasks of inhibition.

Planning and strategy tasks are also typically considered as more cognitively demanding. Gifted children outperform their control peers in geometric problem solving (Vogelaar et al., 2017; Waisman et al., 2016). However, gifted children did not solve the Tower of Hanoi/London planning task with less moves than their peers in control groups (Montoya-Arenas et al., 2018; Vogelaar et al., 2019). This difference in outcomes may be related to varying levels of control within the experimental designs and the differing time constraints during task execution; for example, the relatively unstructured nature of the Tower task allows for more flexibility in the problem-solving process, including variations in time and the strategies employed which could introduce additional confounding variables and make it more challenging to draw definitive comparisons between groups.

A reaction time advantage was demonstrated in 83% of comparisons examining elemental information processing using tasks such as simple reaction time, odd discrimination and abstract matching (Duan et al., 2013; Kranzler et al., 1994). This fact goes in line with our ERP results, which demonstrated shorter ERP latency in gifted children during performance of tasks that require inhibition of irrelevant information (Li et al., 2020; Liu et al., 2011b; Table 6).

Overall, our results demonstrate a consistent advantage of gifted children in verbal working memory, inhibition and geometric problem solving, and shorter reaction time in attentional switching and elemental information processing. Our results align with a recent mini-review that showed the advantages of gifted children in verbal working memory and attentional switching based on the sample of 15 eligible studies (Bucaille et al., 2022). Other researchers (Friedman et al., 2006) specifically showed that working memory is highly correlated with intelligence and showcasing the value of working memory tasks for assessing giftedness, which is also consistent with our results. These findings suggest that verbal working memory and attentional flexibility could be potential cognitive characteristics of giftedness identification. Besides, our results highlight a speed-accuracy trade off pattern, which is mostly prominent in attention and inhibition tasks. We speculate that higher cognitive capacity in gifted children enables them to process the task faster or improve accuracy, particularly in more complex tasks. This mechanism may explain higher reaction time in some tasks and higher accuracy in others in our review. Insights gleaned from psychophysiology data may clarify this trade-off, thereby enriching the discourse on cognitive characteristics associated with giftedness.

### Psychophysiology

In this section, we summarize findings on the psychophysiological characteristics of gifted children which might underlie cognitive characteristics mentioned above. By examining brain patterns, researchers can identify specific regions and networks that are more active or efficient in gifted children. This helps pinpoint the neural basis of advanced skills in problem-solving, memory, and attention.

EEG studies of ERPs show that gifted children differ significantly from the control group in both early and late ERP components. The earliest component of the ERP—P1 (100 ms after stimulus onset)—reflects the earliest stages of information processing. Amplitude of the parietal P1 in response to a stimulus when solving geometric problems was increased in gifted older adolescent boys (Waisman et al., 2016). The authors explain this effect by early analytical activation and more focused attention related to the stimuli in gifted in comparison with control children. Meanwhile, latency of P1 in this study was also higher in the gifted group, which shows a possibly compensatory slower brain reaction in gifted adolescents. However, another study that used the identification of facial expressions task found no differences in P1 between gifted and control younger adolescent boys (Liu et al., 2015). This inconsistency may be explained by the fact that the authors used an affective task, whereas previous studies focused on cognitive tasks, which typically show more stable differences. The absence of differences in P1 for younger adolescents might suggest that they have not yet developed the same cognitive processing capabilities as older adolescents. Furthermore, the differing methods used to identify gifted children—RAPM in one study and Cattell’s culture fair test in another—could contribute to the varying findings across studies.

Articles also examined ERP component N2, which is believed to be related to both classic response conflict (Liu et al., 2011b) and emotional conflict (Li et al., 2020). According to our review, N2 had a larger amplitude in gifted children, which can be interpreted as enhanced conflict processing. The results are consistent between the two studies despite the fact that authors studied different age groups and used different methods for determining giftedness. The latest study by Li et al. (2020) also revealed lower N2 latency in gifted children, which can be interpreted as a faster reaction to the conflict.

Most articles devoted their analyses to a rather late ERP component P3 related to attention allocation processes (Polich, 2007). Most studies revealed increased Р3 amplitude in gifted primary school students and younger adolescents, both in response to a stimulus and in response to a cue (Liu et al., 2011a, 2011b; Zhang et al., 2006), while using different cognitive tasks—from visual search to cognitive control tasks. Also P3 in gifted children had shorter latency (Liu et al., 2011b; Zhang et al., 2006). This is consistent with behavioral findings showing shorter reaction times to cognitive tasks in favor of gifted children. Together, these findings may indicate enhanced and accelerated processes of voluntary or effortful attention processes in gifted children. The increase in P300 during top-down switching was also more pronounced in gifted younger adolescents (Duan and Shi, 2014). Thus, the amplitude and latency of P300 may account for the accelerated reaction times in attentional tasks among gifted children described in the Cognitive ability section.

The N400 is a negative deflection in the ERP waveform of the brain’s electrical activity generally linked with language processing, object recognition, facial recognition, action processing, gesture processing, mathematical cognition, semantic memory, recognition memory, and different developmental and acquired disorders (Kutas and Federmeier, 2011). Our synthesis of past studies showed increased amplitudes in the N400 associated with mental rotation (Anomal et al., 2020) and stimulus discrimination (Liu et al., 2007), presumably reflecting the ability of gifted individuals to devote more cognitive resources to these processes.

In summary, ERP research indicates that gifted children may exhibit more advanced information processing in the later processing stages, such as conflict resolution, top-down attention, discrimination of target stimuli, and mental rotation. Contradictory data have been obtained for the earliest stages of perception. These contradictions might be resolved by considering such experimental factors as age, the specifics of the task, and the method of assessing giftedness. Notice also that gifted children demonstrated increased speed of information processing in the later, more complex processing stages related to attention and conflict resolution, rather than earlier stages of perception (i.e., P1). This may suggest that cognitive differences between gifted children and their peers are more pronounced in complex, late-stage descending processes.

In addition to ERP, EEG activity can be studied by analyzing oscillations obtained using spectral analysis of the EEG signal and reflecting phase-synchronized fluctuations of the membrane potential of neurons (Siegel et al., 2012). Unlike ERPs, studies of EEG oscillations demonstrate not only increased activation related to cognitive processes in gifted children, but also decreased activation. When performing easy tasks for reasoning, gamma rhythm power (30–45 Hz) was lower in gifted older adolescents, which can be interpreted within the framework of neural efficiency hypothesis (Zhang et al., 2015). At the same time, gamma rhythm power increased when the task became more difficult in gifted older adolescents (Zhang et al., 2015). These inconsistent results might also be explained by the relation between task difficulty and the neural efficiency effect and indicate the need to take into account task difficulty in EEG studies of giftedness. It is also important to note that in this study, giftedness was partly determined through mathematical ability. A recent EEG study has shown that when studying the effects of neuronal efficiency, it is important to consider whether math or general giftedness is being assessed (Waisman et al., 2023).

Overall, one could decipher that gifted children demonstrate consistently enhanced and accelerated brain reaction only when performing complex tasks (such as complex reasoning), complex effortful processes (such as cognitive control when processing words) and complex (reflected in late ERP components) stimulus processing processes. This phenomenon might imply the ability to enhance complex information processing that distinguishes gifted children from control children. Our synthesis of results suggests that gifted children demonstrate the ability to activate top-down processing faster and more intensively (excluding at least switching processes). This may indicate an advantage for gifted children in using the limited cognitive resources required to implement this processing. Alternatively, one can entertain that perhaps gifted children have either more resources available or a more optimal functioning of the mechanism for mental effort allocation (Shenhav et al., 2017).

Other indicators of functional brain activity of gifted children in comparison with control children are sparse. BOLD activity in the posterior parietal cortex was increased in gifted older adolescents during a reasoning task (Lee et al., 2006), indicating that superior cognitive ability may stem from enhanced functionality within the fronto-parietal network rather than activation of additional brain regions. Supporting this, Ma et al. (2017) found that gifted children exhibit higher local connection density while relying less on brain hub regions. Furthermore, Nusbaum et al. (2017) demonstrated enhanced inter- and intra-hemispheric white matter integrity in gifted primary school children and young adolescents in frontal, central, and associative pathways, aligning with studies identifying the fronto-parietal network as crucial for intelligence (Dunst et al., 2014; Navas-Sánchez et al., 2014). Additionally, Kimmel and Deboskey (1978) reported that gifted children showed larger initial skin conductance responses and slower habituation compared to average peers, highlighting a connection between autonomic reactivity and intellectual functioning in children.

Overall, the difference in brain activity between the groups is detected only in the late components of evoked potential and in complex top-down processes. This might be explained by the fact that gifted children easily perform better than control children in simple tasks but in order to outperform on complex ones they need to employ additional neural resources. Speculatively, it can be assumed that this brain functioning specificity allows gifted children to outperform their peers in effortful cognitive tasks. However, it is still unclear if it is connected to the greater amount of such neural resources or to their increased motivation in response to the complex task. We will shed some light on this question in the next chapter on psychological characteristics of gifted children.

### Psychological characteristics

In this section, we present findings related to the psychological characteristics of gifted children. A deeper understanding of these traits can provide valuable insights into gifted children’s motivations and self-perceptions, enabling educators to create optimal conditions for unlocking abilities and ensuring mental wellbeing of such children.

Motivation is believed to be highly correlated with giftedness (Lens and Rand, 2000). Some researchers have even included motivation in the definition of giftedness (Gottfried and Gottfried, 2004). Moreover, according to some theories, motivation is a catalyst or resource for the development of giftedness (Gagné, 1985; Sternberg and Lubart, 1993), and low motivation is considered to be the reason for the academic underachievement of gifted children (Whitmore, 1986).

Studies comparing gifted children with a control group have all found higher scores on intrinsic motivation in gifted children (Bergold et al., 2020; Gottfried and Gottfried, 1996; Guez et al., 2018). Intrinsic motivation can be defined as the most self-determined form of motivation, where a student engages in a behavior spontaneously, out of interest and enjoyment. In developmental studies, intrinsic motivation is usually investigated as academic intrinsic motivation, which includes enjoyment of school learning and characterized by curiosity; mastery achievement motivation; persistence in learning; striving for new challenging tasks (Gottfried et al., 2001). External motivation, i.e., exogenous motivation in which a behavior is driven by external factors like encouragement or punishment, is also increased in gifted younger adolescents according to Guez et al. (2018). It is important to note that the causal relationship between motivation and giftedness remains unknown. Moreover, motivation can increase the results of intelligence tests (Duckworth et al., 2011), thus influencing the selection of gifted children. Achievement motivation as an independent construct was also found to be more pronounced in gifted children (Wirthwein et al., 2019). Same results were reported by Gottfried et al. (2006), although the article did not pass the criteria of our review.

Often along with motivation, researchers investigate perceived self-efficacy, a characteristic which can be defined as self-esteem of capabilities in various fields of activity (Bandura and Cervone, 1986). Some authors even include it in the concept of motivation (Schunk and Pajares, 2002), while those who consider self-efficacy a separate personality characteristic have shown that it is increased in gifted children. In particular, this result is observed for academic self-efficacy (Guez et al., 2018), math ability self-concept (Bergold et al., 2020), self-esteem intelligence (Wirthwein et al., 2019), as well as self-regulatory efficacy (i.e., self-esteem of the ability to resist peer pressure to exhibit deviant risk behavior). These consistent results indicate that gifted children highly appreciate their abilities in various areas. It is not clear, however, whether high self-esteem is a consequence or a cause of giftedness. Only for social self-efficacy there was no difference between the groups (Guez et al., 2018). Social self-efficacy was defined as self-esteem of different social abilities. This divergent finding may be explained by López and Sotillo’s (2009) results, which indicated that gifted children did not differ from the control group in measures of social adaptation based on various questionnaires, thus confirming their self-assessment. Speculatively, it can be assumed that enhanced motivation and self-efficacy are related to the specificity of effort allocation mechanism that allow gifted children to outperform their peers in effortful cognitive functions.

Studies using the Big 5 factors found that gifted adolescents were significantly more Open to experience and did not differ from their peers in Extraversion, Agreeableness and Conscientiousness (Limont et al., 2014; Wirthwein et al., 2019). The authors suggested that openness as the desire for new experience might be necessary for the development of giftedness. However, it is important to note that the results obtained do not indicate whether the revealed psychological characteristics of gifted children are a consequence or a cause of giftedness.

In summary, motivation seems to be consistently associated with giftedness, with gifted children showing higher levels of both intrinsic and extrinsic motivation, as well as enhanced self-efficacy—except for social self-efficacy. Gifted adolescents are also more open to experience.

To summarize the reviewed studies on the distinctive cognitive, psychophysiological, and psychological characteristics of gifted children, we highlight their unique traits across various domains. Our findings suggest a general advantage of gifted children in either reaction time or accuracy across key cognitive domains, including verbal working memory and attentional switching but also inhibition, geometric problem solving and elemental information processing. This advantage aligns with enhanced and accelerated brain activity during complex effortful processes, presumably due to their greater availability of brain resources or higher motivation in gifted children. Our findings further support the notion of generally higher intrinsic motivation in gifted children in comparison with their peers in the control group. Additionally, our investigation revealed heightened self-efficacy and openness to experience in gifted children as well as higher school achievements and different problem-solving strategies.

The importance of these results comes both from insights gained in basic research by uncovering characteristics of giftedness, and from the contribution these findings may make to the development of educational programs tailored for gifted individuals. Advantage in reaction times and accuracy across specific mentioned cognitive domains can aid in developing more targeted assessment methods for children’s abilities. The observation that gifted children exhibit enhanced brain activity during complex tasks but roughly equivalent one in comparison with their peers during simple tasks, might suggest to educators that the most developmental environments for gifted students are those that provide increased intellectual challenges. Additionally, by examining the brain patterns of gifted children in general, researchers can clarify the underlying mechanisms behind high intellectual potential. Gifted children’s heightened intrinsic motivation, self-efficacy, and openness to experience can offer educators valuable insights into creating environments that support their potential and wellbeing.

In the next section we elaborate on the future research directions to advance the state of the art in the field of intellectual giftedness.

### Considerations for future studies

#### Cognitive function terminology and measurement variability

The review of the literature and the generalized interpretation of the results of research are greatly hampered by inconsistent terminology describing cognitive functions. Different authors rely on different theoretical models of cognitive abilities, which gives rise to an unlimited number of studied functions and their terms. We recorded approximately 50 different terms that describe higher top-down information processing (see column ‘Target of analysis’ in Supplementary Table 1S). Most terms denoted some form of attention, both in the broadest sense (as cognitive control or executive functions), and its various aspects (divided, selective, spatial, etc.). Many authors have also studied higher-level cognitive abilities, such as reading, abstract reasoning, mental rotation, etc. It was often difficult to decipher specific semantic differences among studied functions, for example, between analogical and abstract reasoning, attentional control and cognitive control. To mitigate this limitation, we used terminology proposed by original articles. We also encourage future studies to add a short paragraph outlining how the terminology they use relates to others in the field (Table 11, step 1).

**Table 11:** Seven step checklist for studying outstanding performers.

Step	Title	Description
Step 1	Match terminology	Relate terms used in the current study with one or more constructs in the literature
Step 2	Selection criteria	Provide rationale for assessment tools, metrics and score criterion for group selection
Step 3	Sample	Provide recruitment details and sample demographics in terms of country, age, gender, etc.
Step 4	Paradigm details	Provide paradigm details for all assessments used that include task instruction, task timing, parameters recorded
Step 5	Statistical significance	Provide the threshold used for statistical significance (not applicable to Bayesian statistics)
Step 6	Correlational analysis	Consider performing correlational analysis
Step 7	Availability of data	Make the data available for download in an online repository.

To examine cognitive functions of gifted and control children a wide variety of tasks was created by researchers. Variability in measurements of cognitive abilities complicates the feasibility of conducting a quantitative meta-analysis due to the heterogeneity of data. Further, the use of complex tasks that activate several cognitive mechanisms at the same time might complicate the comparison between the studies. Although not always possible because of time and resource restrictions it is advisable to include well-established cognitive tasks that can build the literature for ultimately identifying convergence across studies using quantitative meta-analyses.

#### Lack of standardization in methods

The field “Other tests” takes the second position in the list of widely used test to identify giftedness in children (Figure 2). This category includes all the validated tests which were used for selection. The profusion of tests, even those with established credibility, could potentially impact the coherence among them, further making interpretations more challenging. The use of thresholds in different studies to define giftedness varies significantly, reflecting differences in assessment tools, age groups, and research objectives. This lack of standardization suggests a need for more consistency and clarity in defining and measuring high intelligence in research and educational settings. To ultimately reach consensus we propose that future studies provide a rational for assessment, metrics and score criteria used to classify groups (Table 11, step 2).

#### Binary group division

We only analyzed data from studies that compared psychological measures between the groups of gifted and control children. However, the idea of dividing participants into two groups may be controversial in its origin. Though, it is necessary to determine the cut-off point when selecting gifted children to study in a gifted class, for research purposes there is no clear ground for such division as linear regression models are always available to identify the relation of giftedness with other characteristics of participants. Besides, categorizing intelligence through precise numerical divisions is somewhat contrived, given that intelligence spans a broad spectrum. Therefore, we suggest that future studies consider correlational approaches or organizing three or more groups specified criteria (Table 11, step 6).

#### Reporting style

The variability in the reporting styles of the studies also affects the result of systematic reviews and meta-analyses. Missing indication on the method of selection (or unclear definition), age and number of participants, and task descriptions limits the ability to conduct comprehensive and reliable syntheses of existing research. Variability in reporting style also has several implications on research in general. It may hinder the ability of other researchers to replicate the study accurately. Besides, inconsistencies in reporting make it challenging to compare results across different studies and may impact the generalizability of study findings. For these reasons, in our seven steps for studying outstanding performers we propose that future studies clearly define sample recruitment details and demographics, provide details paradigm administration, report statistical significance thresholds and tabulate descriptive statistics.

In summary, current research on gifted children reveals notable methodological, conceptual, and reporting gaps. These include variations in measurement techniques, a lack of standardization in assessment methods, contentious binary group classifications, inconsistent terminology for cognitive functions, and disparities in reporting styles, all of which impede reliable comparisons and synthesis of findings.

## Limitations

The review has some important limitations. First, the study included search only from one database Web of Science due to resource constraints. Web of Science is one of the most popular and widely used databases for academic research and publication, which is known for its interdisciplinary coverage. Web of Science is also recognized for hosting high-impact journals and authoritative sources in gifted education and intelligence fields. Prior to finalizing our database selection, we conducted preliminary scoping searches in databases Web of Science and PubMed databases. The search in PubMed revealed substantial overlap with the articles indexed in Web of Science and did not yield a significant number of additional unique studies relevant to our topic. Therefore, we determined that Web of Science alone was sufficient to capture the breadth of literature necessary for our review. Nevertheless, we acknowledge that limiting our search to single database might bias the coverage of the relevant literature. Second, we acknowledge that restricting our review to English-language articles may introduce language bias, limit the comprehensiveness of our findings and affect the generalizability of our conclusions. Future research could benefit from including non-English studies or collaborating with multilingual researchers to provide a more exhaustive overview of the literature.

Third, the study includes only dichotomized information (gifted or non-gifted children), thus ignoring literature that examined correlation between constructs. We acknowledge that this fact diminishes the variability in the data and makes the results harder to interpret, especially with the differing cutoffs across studies. However, in the educational context identification of talented children is always a practical task: children either meet the criterion established by a specific program or not. Thus, in the current systematic review we rely on binary group allocation.

Forth, all the eligible studies were given the same weight when summarizing the results. It was done to ensure that a diverse range of perspectives and findings are considered in the review, which is particularly relevant as the research of giftedness is broad and there is limited consensus in the literature. Assigning equal weight might also help to mitigate potential biases that could arise from selectively emphasizing studies with larger sample sizes or more complex methodologies. This approach promotes a more balanced representation of the available evidence. Nevertheless, giving equal weight to all relevant papers may potentially affect the overall robustness of the review findings. In addition, the review did not analyze effect sizes of the included studies, since in many articles it was not indicated or was calculated by different metrics.

## Conclusion

This systematic review has provided a synthesis of the prevalent intelligence tests in identifying intellectual giftedness in children and the clearer understanding of cognitive, physiological and psychological characteristics which distinguish gifted individuals from their control peers. Our findings delineate a noticeable shift from traditional intelligence tests toward the utilization of more culturally appropriate measures. Comparisons among gifted and control children reveal superior abilities of the former ones in verbal working memory, inhibition, geometric problem solving, increased speed in attention-switching and elemental information processing. Consistently, psychophysiological assessments demonstrate heightened and accelerated brain activity during effortful cognitive processes. Psychological and behavioral tests further demonstrate that gifted children score higher on tests measuring intrinsic motivation, self-efficacy, and openness to experience, as well as achieving higher grades in school and employing better problem-solving strategies. We propose a simple seven step checklist for studying outstanding performers as our review emphasizes the need for continued research and refinement in assessment methodologies to advance the state of the art in the field of intellectual giftedness and address the unique needs of gifted children in educational context.

## Data Availability

The original contributions presented in the study are included in the article/Supplementary material, further inquiries can be directed to the corresponding author.

## Footnotes

^1^ webofknowledge.com
